# Population encoding of observed and actual somatosensations in the human posterior parietal cortex

**DOI:** 10.1073/pnas.2316012121

**Published:** 2024-12-30

**Authors:** Srinivas Chivukula, Tyson Aflalo, Carey Zhang, Emily R. Rosario, Ausaf Bari, Nader Pouratian, Richard A. Andersen

**Affiliations:** ^a^Department of Neurological Surgery, UT Southwestern Medical Center, Dallas, TX 75390; ^b^Department of Biology and Biological Engineering, California Institute of Technology, Pasadena, CA 91125; ^c^Tianqiao and Chrissy Chen Brain-Machine Interface Center, Chen Institute for Neuroscience, California Institute of Technology, Pasadena, CA 91125; ^d^Casa Colina Hospital and Centers for Healthcare, Pomona, CA 91767; ^e^Department of Neurological Surgery, University of California, Los Angeles, CA 90095

**Keywords:** internal Model, generalizability, compositionality, mirror neurons, posterior parietal cortex

## Abstract

Cognition relies on transforming sensory inputs into a generalizable understanding of the world. Mirror neurons have been proposed to underlie this process, mapping visual representations of others’ actions and sensations onto neurons that mediate our own, providing a conduit for understanding. However, this theory has limitations. Here, we hypothesize that mirror-like responses represent one facet of a broader framework in which our brains engage internal models for cognition. We recorded populations of single neurons in the human posterior parietal cortex (PPC) of a brain–machine interface clinical trial participant implanted with a microelectrode array while she either experienced actual touch, or observed diverse tactile stimuli applied to other individuals. Two body locations were tested, on each of the participant and other individuals. Some neurons exhibited mirror-like properties, consistent with earlier literature. However, they were fragile, breaking with increased task complexity. Population responses were better characterized by generalizable and compositional basic-level features encoded within neural subspaces. These features enable the population to respond to diverse actual and observed touch stimuli and are recruited similarly for similar forms of touch. Mirror-like neurons belong within these subspaces, contributing more globally to compositionality and generalizability. We speculate that at a population-level, human PPC manifests an internal model for touch, and that cognition unfolds in the high-level human cortex by versatility in its representational building blocks. In a broad sense, we speculate that the population features we demonstrate support a broad mechanism by which the high-level human cortex enables understanding.

## Significance

We show that in humans, neurons in the posterior parietal cortex (PPC) store basic-level information regarding different forms of touch (e.g., pinch, press, rub, tap) to different body locations that can combine in myriad ways (a property known as compositionality) and be recruited to diverse situations (known as generalizability). We then demonstrate a versatile mechanism by which the shared population-level substrate can recruit these representational building blocks of touch to construct similar but distinct responses to many forms of touch we observe to others, or actual touch to ourselves. Our report is thus significant for mechanistically describing how knowledge in the human PPC can contribute to our understanding of experienced and observed somatosensations.

We do not just see the world, we understand it (1–3). From a brief video or even a still image of a person in action, we can infer what they are doing, why they are doing it, what they will do next, or what they might have done but did not. A fundamental question in neuroscience is how neural populations transform sensory inputs into such deep and versatile understanding.

Mirror neurons have been proposed to be the neural basis for how we understand what another person intends or feels (2, 4). In this view, we map the visual representation of others’ actions, emotions, or sensations, onto our own corresponding neurons and thereby attain understanding (2, 4, 5). However, this hypothesis has received numerous critiques (6, 7). For example, if understanding comes from activating our own high-level action representations, how can we understand actions we have never performed (e.g., jumping a skateboard)? Or could never perform (e.g., flying)? Further, mounting literature including recent multielectrode array recordings in high-level motor regions of the human cortex have emphasized that single-neuron behavior is complex; some neurons exhibit specificity and congruency, firing only to a particular action or emotion by oneself or another, while the majority activate more indiscriminately (8–10). To date, a mechanistic view for how heterogenous populations of neurons within the high-level cortex that include mirror neurons collectively enable understanding has remained elusive.

Alternate theoretical frameworks have emerged in parallel based on the idea that human-like learning and thinking is the product of how neural systems build and use internal models of the world, what we will call the “cognition through internal models” framework (3, 11). Internal models are our brain’s representations of ourselves and our physical world: the objects in it, how they interact, and how they do not. The power of these internal models lies in its defining features of generalizability and compositionality. Generalizability captures the idea that representations apply across contexts and behaviors, providing a common substrate to inform our perception, cognition, imagination, and planning across many situations. Compositionality captures the idea that multifaceted representations are constructed as a combination of more basic-level representations (12, 13). Recent studies have demonstrated compositional encoding at the neural population level (8, 14–17) suggesting that a similar scheme might underlie neural responses including those associated with mirror neurons. Such an encoding architecture is highly versatile and may provide a broader framework for human cognition within which mirroring may represent one manifestation.

We have recently recorded populations of single neurons in the human posterior parietal cortex (PPC) during motor, sensory, and cognitive behaviors. These neurons encode many diverse body-related variables such as action verbs, observed actions, motor and sensory imagery, and motor plans (9, 10, 18, 19). Individual neurons are often complex, yet population-level representations demonstrate generalizable encoding across these varied domains in a functional organization we termed partially mixed selectivity (10). Based on these past results, we hypothesize that human PPC encodes a general mechanism by which we create generalizable internal representations of the world (20). To test this hypothesis, we recorded from populations of neurons in human PPC while a participant experienced actual touch (to the participant) or observed touch (to another individual). We found that a small number of neurons exhibited mirror-like responses, firing in similar manners to similar forms of actual and observed touch, consistent with earlier literature. However, across the different forms of touch stimuli tested, population-level neural responses were more consistent with encoding generalizable and compositional basic-level tactile variables related to body location and touch type, like representational building blocks embedded within latent neural subspaces. These variables are recruited for diverse forms of actual and observed touch in a context-dependent manner, providing a mechanism for shared representations to emerge at a population-level within human PPC. Based on these findings, along with our lab’s earlier discovery that this same human PPC substrate also encodes many different types of actions (e.g., attempted, imagined, executed), we speculate that human PPC encodes mutually shared and versatile basic-level features that it recruits as necessary across domains and across contexts. Such a view is consistent with the cognition-through-internal-models framework, while also accounting for the mirror-mechanism.

## Results

We recorded populations of single neurons from a microelectrode array implanted in the PPC of participant NS, a tetraplegic individual (spinal injury at cervical levels 3 to 4; C3/4) participating in a clinical brain–machine interface study (*SI Appendix*, Fig. S1). Neurons in this region had well-defined tactile receptive fields that responded at short (~60 ms) latencies consistent with bottom–up sensory processing (19). Such responses opened the possibility of studying whether, and how, these PPC neurons support mirror-like phenomena. We performed two primary experiments: The first used a simple paradigm to confirm mirror-like properties; the second expanded the number of task dimensions to determine how shared yet distinct representations in diverse sensory contexts arise within populations of human PPC neurons.

### Some Single Neurons in Human PPC Exhibit Mirror-Like Responses.

We found many compelling examples of neurons with mirror-like responses. Movie S1 shows an example neuron demonstrating specificity and congruency. Specificity is the selective activation to a restricted set of tactile stimuli, evidenced by the neuron responding to touch to her outer shoulder but not her inner shoulder. Congruency is defined as having similar neural responses when experiencing or when observing the same tactile stimulation. Movie S2 shows an additional example.

We note that over the years “mirror neurons” have sometimes been used for neurons with highly congruent activity during execution and observation of movement, and at other times more widely for all neurons modulated during both execution and observation even if incongruent.(21–24) Some groups have gone as far as to categorize mirror neurons into three broad classes: “strictly congruent,” “broadly congruent,” and “noncongruent.”(25) For this manuscript we use a strict definition of high congruence when using the term mirror neuron, or describing their properties.

**Fig. 1.**
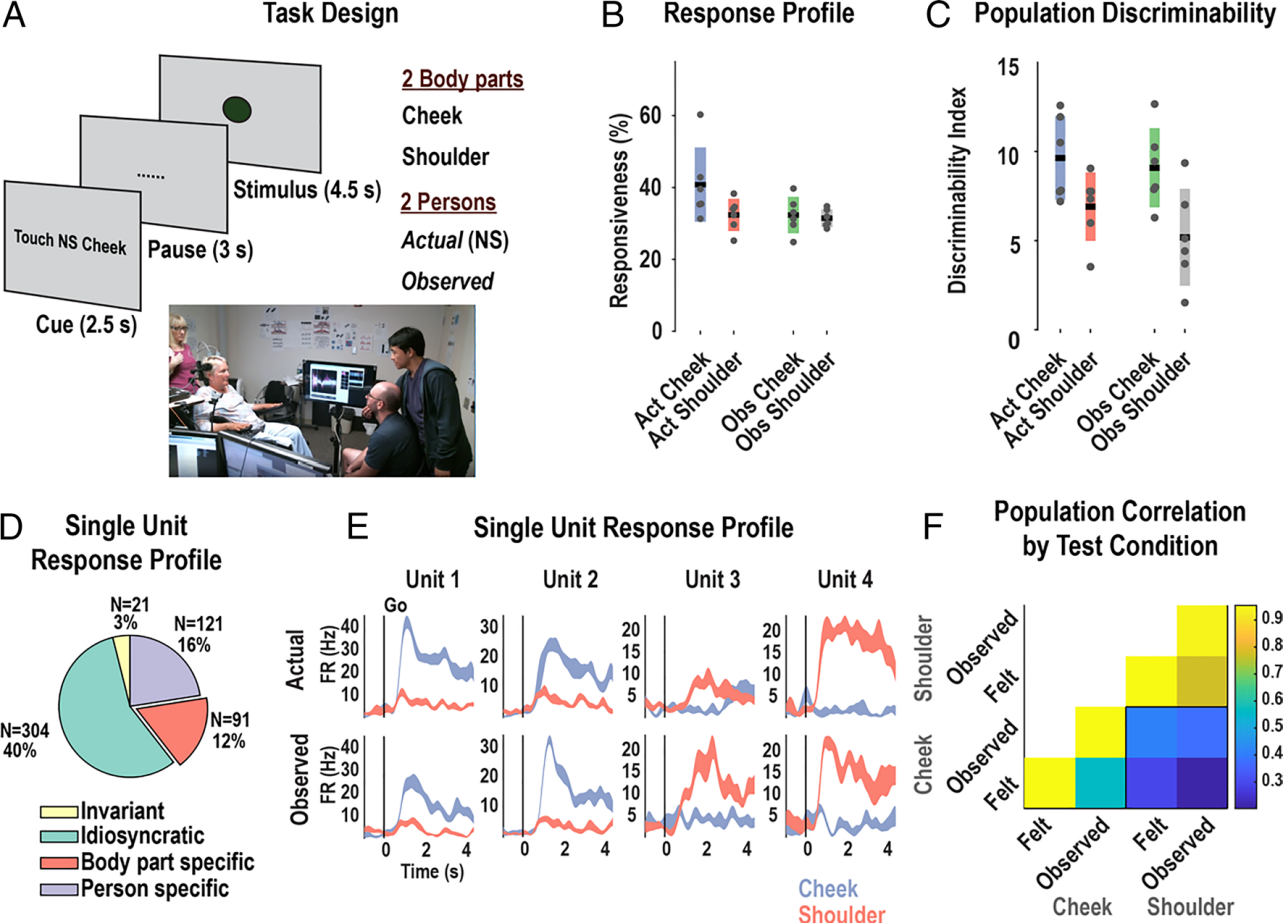
Evidence for mirror-like responses for actual and observed touch in human PPC. (*A*) Task design testing neural responses during actual and observed touch to the cheek and shoulder. The cue (hidden from the participant) instructed experimenters on which tactile stimulus to deliver during the stimulus phase. (*B*), Percent of neurons demonstrating significant modulation from the inter-trial-interval baseline (*P* <0.05, FDR corrected, mean ± 95% CI, 10 trials per condition, 757 neurons). Gray dots represent single-session results. The bars show the mean (horizontal black line) ± 95% CI computed across sessions. Act = actual; obs = observed. (*C*) Population measure of the strength of representation as measured by the distance of neural population response from the baseline ITI period baseline (Mahalanobis distance, mean ± 95% CI across sessions). Gray dots represent single-session results. The bars show the mean (horizontal black line) ± 95% CI computed across sessions. (*D*) Pie chart categorizing neurons according to their individual response properties: body part specific (invariant to whether touch was actual or observed, i.e. mirror-like), person-specific (invariant to body part), invariant (responsive to all conditions), or idiosyncratic (other patterns). (*E*) Example neurons showing mirror-like responses (mean±SEM, n = 10 trials.) Each column shows the response for one neuron to actual (*Top* row) and observed touch (*Bottom* row.) (*F*) Cross-validated correlation of population responses within and between conditions. Colors represent the correlation strength, as in the scale.

We performed a basic sensory mirroring task (Experiment 1 – BSMT, Fig. 1*A*) to quantify the existence of sensory mirror-like responses. We recorded an average of 126 ± 20 neurons over 6 sessions while the participant felt rubbing motions applied to her cheek or shoulder or observed rubbing motions applied to an experimenter’s cheek or shoulder. This two-factor (body part x person) design allowed us to test for specificity and congruency. We found robust coding of actual and observed tactile sensations (Fig. 1 *B* and *C*). Many neurons demonstrated mirror-like responses, firing similarly to touches to the cheek or shoulder (specificity), invariant to whether the touches were actual or observed (congruency) (model analysis, body part specific, *P* <0.05 corrected, Fig. 1*D*). Example neurons showing mirror-like responses are shown in Fig. 1*E*. We summarized the response of the entire population during each condition as a vector of the mean firing rates while the participant experienced actual touch or observed touch. We found that population correlations between conditions were higher for matching body parts than for mismatched body parts (*t* test, *P* <0.05, Fig. 1*F*). Thus, mirroring behavior was robust at the neural population level. However, from this simple paradigm, it is unclear how populations of PPC neurons store information in a way that it can be manipulated to support diverse touch responses, including shared representations (or mirroring behavior) across similar contexts of actual and observed touch. Tellingly, only 12 percent of neurons demonstrate specificity and congruency, consistent with mirror-neuron literature in which mirror neurons have been noted to constitute only a small fraction of heterogenous populations of neurons.

### How Does PPC Mediate Similar Coding of Actual and Observed Sensations?

To better understand similar encoding between actual and observed sensations, we performed a second experiment (Experiment 2) that augmented the first experiment to include four different types of touch (pinch, press, rub, and tap). These touch-types were selected as they resulted in perceptually distinct stimuli under observed and actual touch conditions and not based on assumptions about the underlying selectivity of recorded neurons. Thus, in the updated task, three manipulated dimensions (body part, touch type, and person) are combined in a full factorial design for 16 total conditions. Including the additional dimension allowed us to 1) test how well the mirror account scales to more complex stimuli; 2) whether shared encoding is particular to self and other, or a more ubiquitous property of the neural population; and 3) whether these responses are consistent with a compositional basis, encoding multidimensional sensations as a combination of basic sensory properties. We recorded an average of 119 ± 16 neurons over 8 sessions.

### Single-Neuron Responses Are Highly Complex and Variable and Cannot Explain the Population Response.

**Fig. 2.**
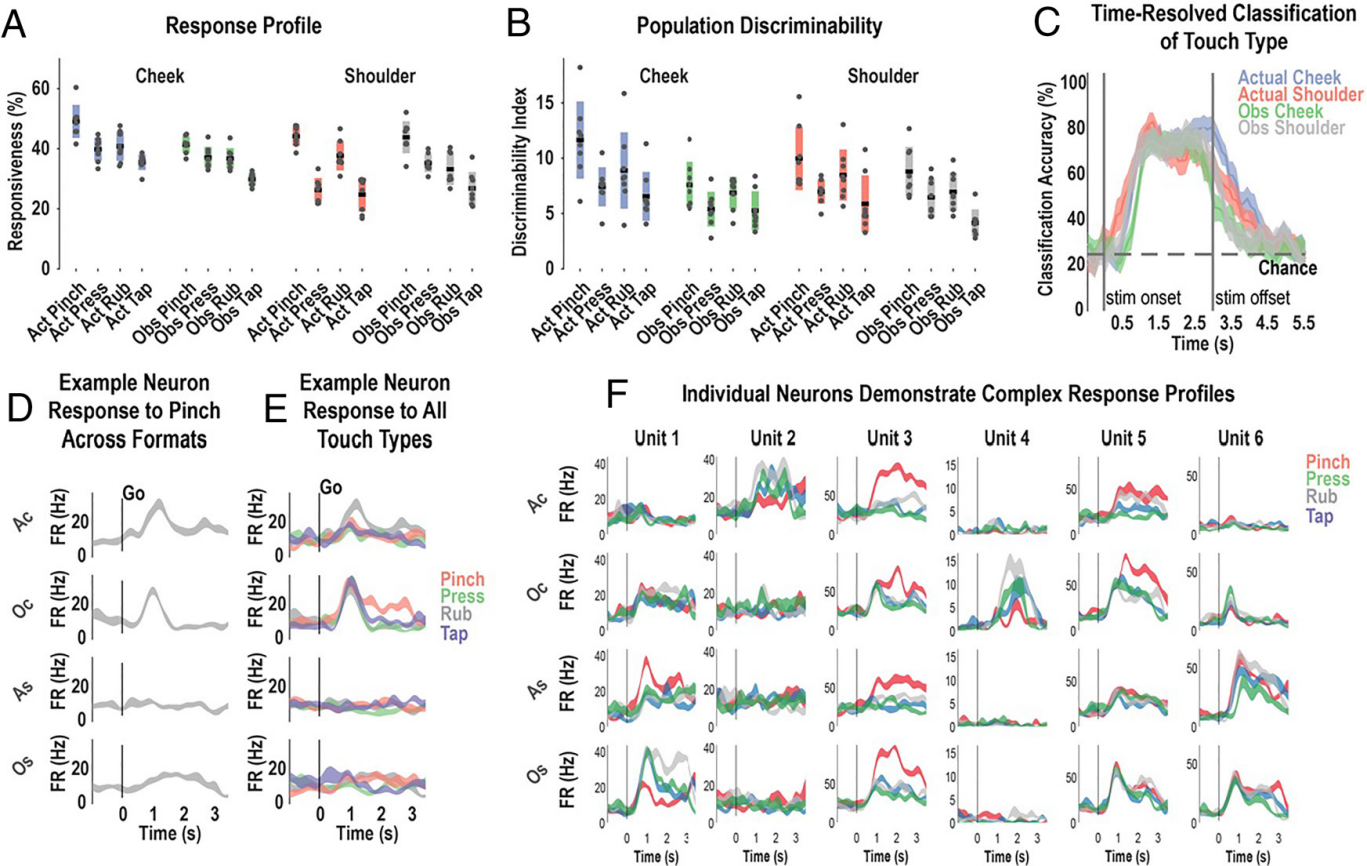
Single neurons discriminate many types of actual and observed touch. (*A*) Percent of neurons demonstrating significant modulation from the inter-trial-interval baseline (*P* <0.05, FDR corrected, mean ± 95% CI, 10 trials per condition, 757 neurons). Gray dots represent single-session results. (*B*) Population measure of the strength of representation as measured by the distance of neural population response from the ITI period baseline (Mahalanobis distance, mean ± 95% CI across sessions). Gray dots represent single-session results. (*C*) Time-resolved, cross-validated classification accuracy discriminating the four touch-types within each sensory field (mean ± 95% CI computed across sessions). (*D*) Sample neuron response to pinch across all sensory fields as a function of time (mean ± SEM, n = 10 trials.) (*E*) Response for the same example neuron from panel *D* across the four sensory fields, now including all touch types. Touch types are color-coded, as indicated. Other details are as in panel *D*. (*F*) Additional example neurons (*SI Appendix*, Fig. S2). Each column depicts the response for one unit to each sensory field (rows). Details as in panels *D*. Act, actual; Obs, observed; s, seconds; Ac, actual cheek; Oc, observed cheek; As, actual shoulder; Os, observed shoulder; Hz, hertz.

As in the first experiment, we found robust coding of actual and observed tactile sensations (Fig. 2 *A* and *B*). The response to the different touch types could be discriminated for actual or observed conditions (time-resolved classification, Fig. 2*C*). However, the inclusion of additional touch-types highlighted the near-universal complexity of single-unit responses: Neurons that appeared to have a simple mirror response for a single touch-type were no longer easily reconciled with a mirror neuron account: Fig. 2*D* shows a neuron that responds similarly to actual or observed pinches to the cheek, but not the shoulder, consistent with a mirror account. However, testing the same neuron with additional touch-types reveals a more complicated pattern (Fig. 2*E*): The neuron is selective for pinches to her own cheek but responds to all touch-types during observation. A straightforward interpretation of the mirror mechanism would predict that NS would understand all touch-types as a pinch, inconsistent with behavioral evidence that the touch types were easily discriminated and the finding that observed touch types are discriminable (e.g., Fig. 2*C*). Additional example neurons illustrating heterogeneous and complex responses are shown in Fig. 2*F* and *SI Appendix*, Fig. S2 (*SI Appendix*).

**Fig. 3.**
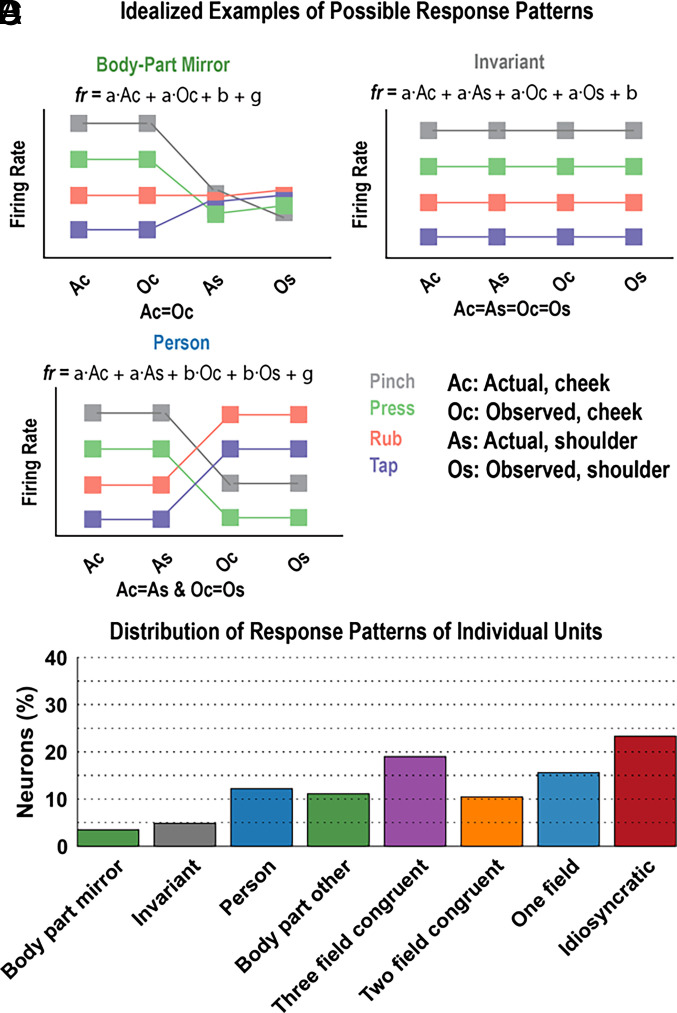
Single neurons are complex and heterogeneous. (*A*–*C*) Schematic illustrations of three of the 51 possible linear models describing how a neuron’s firing rate response to the four touch-types (selectivity pattern, SP) is congruent or incongruent across the four sensory fields (Ac, As, Oc, Os; see legend). A more complete description can be found in *SI Appendix*, Fig. S3. (*D*) Histogram showing the percentage of PPC neurons that behaved according to each category of models (*SI Appendix*, Figs. S3–S5). Ac, actual cheek touch; As, actual shoulder touch; Oc, observed cheek touch, Os, observed shoulder touch; fr. firing rate.

We used a model selection analysis (9) to categorize patterns of congruency across all sensory fields (e.g., the cheek or shoulder, on NS or the experimenter). For each neuron, we fit linear tuning models that described the response of the neuron to the four touch-types (selectivity pattern, SP) as either congruent or incongruent across sensory fields. There are 51 such possible models (*SI Appendix*, Fig. S3). Three schematic examples illustrating congruency patterns are shown in Fig. 3 *A*–*C*. From among the 51 possibilities, we identified the linear model that best described neural behavior using two metrics: Bayesian information criterion (BIC) and cross-validated coefficient of determination (cvR^2^). The percentage of PPC cells that behaved according to each model is shown in *SI Appendix*, Fig. S4. Fig. 3*D* summarizes the result by grouping responses into eight general categories that captured high-level modes of behavior (see *SI Appendix*, Fig. S5 for the results split by BIC or cvR^2^ criteria). A summary description of the eight modes can be found in the legend of *SI Appendix*, Fig. S3 (*SI Appendix*).

The PPC population was heterogeneous, composed of many complex patterns of congruency across sensory fields. With the inclusion of the additional touch-types, only 3% of neurons show specificity and congruency for coding the body location that was touched (compared to 12% in the simple task, Fig. 1*D*). This result highlights the fragility of single unit mirror responses as we expand the paradigm to include a broader diversity of stimuli.

### The Architecture of Knowledge Representation in Human PPC is Consistent with an Encoding of Compositional and Generalizable Features of the Stimuli.

Traditional mirror neuron studies have typically categorized a particular neuron as a mirror neuron if it fired identically to a particular action (e.g., grasp) whether executed or observed. Our unique setup of being able to record from a multielectrode array in a human participant allowed us to relatively easily test the responsiveness to many forms of touch simultaneously. With increasing task dimensions, we found that neurons within human PPC exhibited significant complexity. We hypothesized that complex single-unit behavior is at the core of a compositional and generalizable population encoding scheme that enables NS to build an internal model of the tactile world. In other words, at a population level, the scene is broken down into its most primitive elements, such as what part of the body is being touched and how it is being touched. The neural representation of these primitives can be learned from data. Further, these building blocks must be universal, in the sense that they can used to understand both what NS is feeling as well as what she is seeing. Thus, the building blocks that are extracted in one context should also apply to other contexts. Such a line of thinking has proven fruitful in understanding how we build internal representations of faces (17), encode information about timing and task variables (14), or code movement across multiple effectors (8). We wished to confirm the representation of such building blocks in human PPC for touch.

**Fig. 4.**
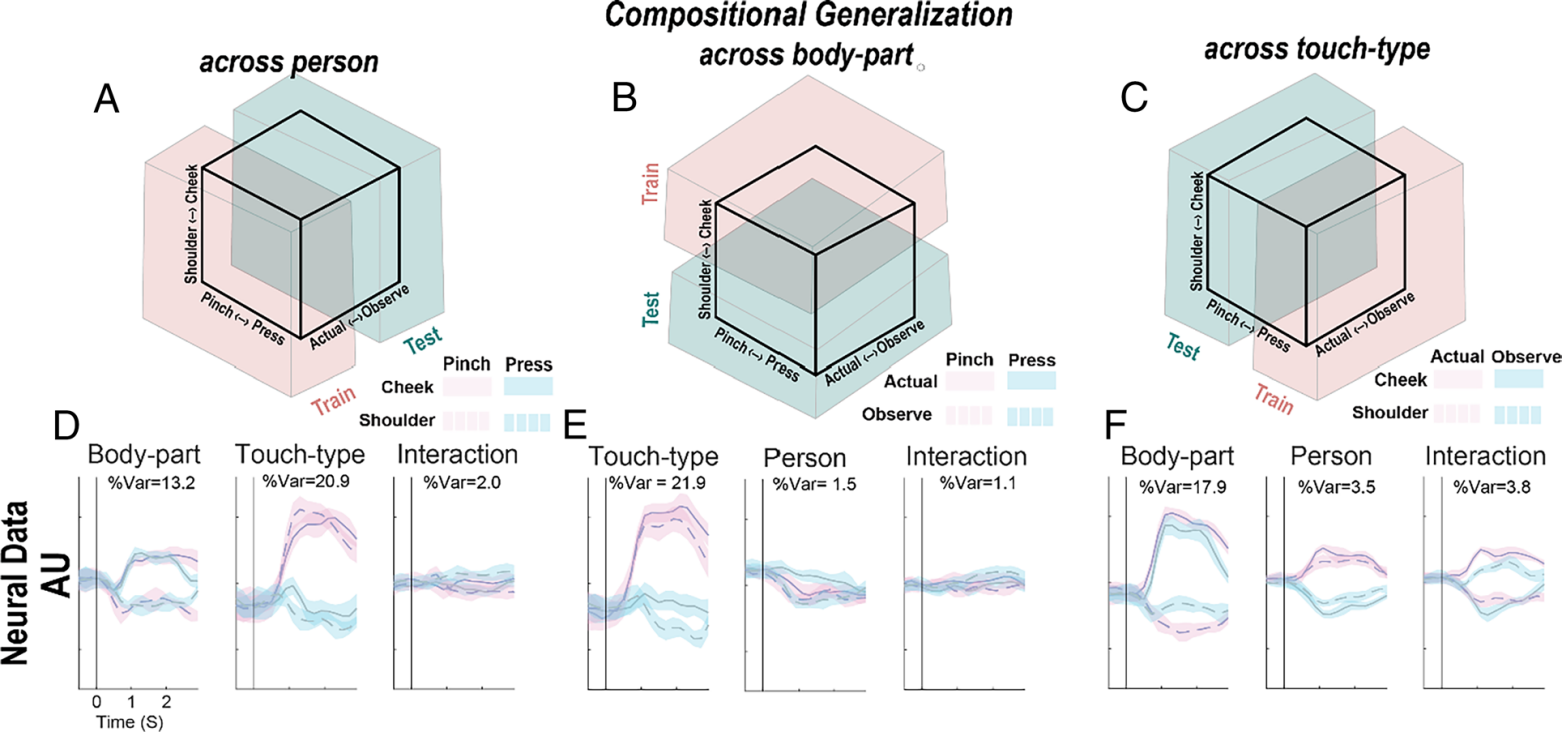
Human PPC encodes actual and observed touch using a generalizable and compositional encoding scheme. (*A*) Schematic illustration of testing the generalizability of compositional coding of touch-type and body-part across actual and observed touch. The training data are used to find a linear mapping that explains the population response as a combination of latent variables related to touch-type and body-part. The generalizability of these subspaces is then validated by testing whether the learned mapping applied to the held-out test data can recover these subspaces. (*B* and *C*) Similar to *A*, for different splits of train and test datasets. (*D*) Characterizing the generalization of learned latent subspaces. Each panel visualizes the projection of the test data onto the latent neural subspaces of interest (see panel titles) identified in the training data. Within the latent space, the condition-labeled (see legend) test data separate and preserve encoding of the basic-level tactile variables. Results are shown as the mean ± 95% CI across sessions, as a function of time (x-axis). (*E* and *F*) Same analysis as in *D*, except performed on train-test splits defined across body-part and touch-type respectively.

To test for generalizable compositional encoding, we took advantage of our experimental design that varied stimuli across three behaviorally meaningful dimensions (touch-type, body-part, and person). Our goal was to identify putative building blocks using a training set composed of two of these dimensions using a single level of the third dimension and validate the generalizability of this coding using the second level of the third dimension. This scheme is schematically illustrated in Fig. 4*A*: The training data are composed of neural data collected during actual touch and the test data are composed of data collected during observed touch. The training data were used to find a mapping that described the population behavior as a linear composition of latent representations related to touch-type and body-part (e.g. our so-called building blocks). This learned mapping was applied to the test data to ensure that the identified latent dimensions were sufficient to explain the neural behavior in the new context (e.g. providing evidence that the building blocks are generalizable). This process was repeated for the other ways of partitioning the data (Fig. 4 *B* and *C*).

Fig. 4*D* shows the results of our analysis when testing generalizability across actual and observed touch. Each panel illustrates the temporal evolution of latent dimensions related to body-part, touch-type, and their interaction respectively. As hypothesized, the latent representations of the basic touch variables were able to explain the behavior of the held-out test data: When the test data were projected into, e.g., the putative latent space that defines touch-type, we found that the different touch types were well separated. In other words, we were able to identify basic building blocks and these building blocks generalized across contexts thus supporting the hypothesis that the neural population supports compositional coding of basic-level touch variables. The nonlinear interaction did not generalize, suggesting that the population maintains a distinct representation of these touch variables. Critically, Fig. 4 *E* and *F* further demonstrate that latent representations of touch-type and body-part generalize across all splits of the data supporting the hypothesis that these variables are fundamental aspects of the population code that generalize across multiple contexts, not just self and other. In contrast, we found that the person variable did not always generalize or explained only a small portion of the population variance. This may reflect a broader organization where different types of variables are localized to different regions of the cortex (*Discussion*).

**Fig. 5.**
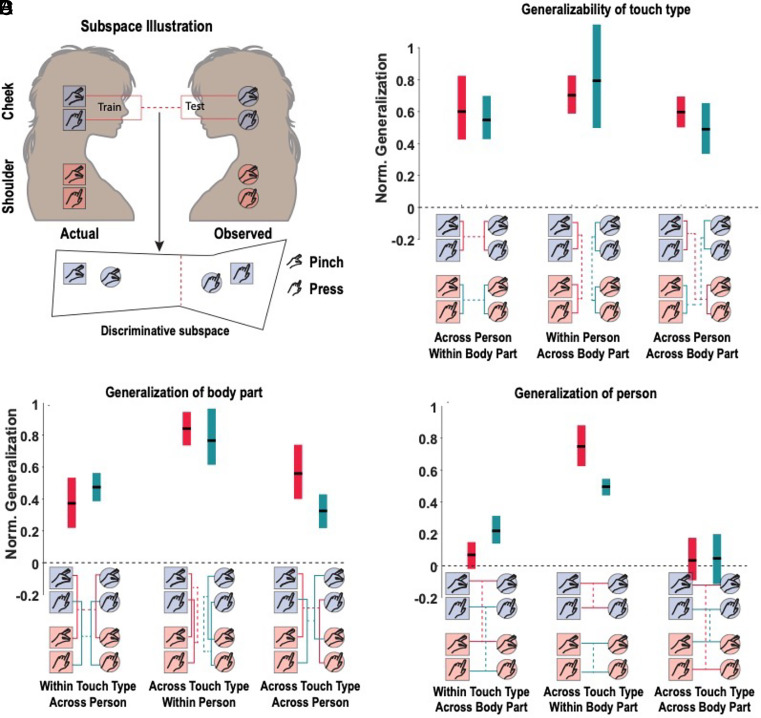
Population-level subspaces mediate the generalization of tactile information across all task dimensions. (*A*) Schematic illustration of the subspace analysis to test the generalizability of tactile information. In this example, we learn a linear mapping that discriminates actual pinch and press to the participant’s cheek from population neural activity. We then test how well the mapping is able to discriminate data collected while the participant observes pinch and press to the experimenter’s cheek. Generalization is quantified by measuring the Mahalanobis distance between conditions in the observed (test) data normalized by the distance between conditions in the actual touch (training) data. (*B*) Results of the subspace analysis when testing how touch-type information generalizes across the other two dimensions: body part and person. The normalized generalization (*y*-axis) is shown for each tested subspace (*x*-axis). On the x-axis below each group of bars is a condensed schematic (from panel *A*), showing the train and test pairs. The red and green lines illustrate two separate but related tests for generalizability. The bars show the mean generalization (horizontal black line) ± 95% CI computed across sessions. (*C*) Similar to panel *B*, except here the generalizability of body part information is being tested across touch type and person. (*D*) Similar to panels *B* and *C*, except here the generalizability of person information is being tested across body part and touch type. Norm, normalized.

In a complementary analysis, we looked at a population-level generalization of the basic mirror neuron test, testing for both specificity (operationalized by finding the population response that discriminates between two conditions) and congruency (testing whether this population response is congruent between self and other, or potentially other task dimensions). We find comparable congruency across all task dimensions, supporting a model of generalizable encoding in this population of PPC neurons (Fig. 5).

## Discussion

### Shared Representations Across Actual and Observed Touch in the Human PPC.

Mirror neurons are the foundation of an influential theory for how we understand the actions and experiences of others (2). According to the mirror hypothesis, neurons within high-level regions responsible for planning our own motor behavior or processing bodily sensations or emotions are also involved in understanding the intentions and experiences of others. Our data are consistent with this view.

However, true mirror neurons only account for a small fraction of neurons within the brain regions they have been found in, both in nonhuman primates, and more recently, in humans (8, 10, 26–29). Individual neurons and population-level activity in these regions are often more complex. A growing literature indicates that mirroring-like behavior is mediated by populations of neurons with contributions from neurons that do not directly exhibit the congruency associated with classic mirror neurons (24, 29–31). Moreover, most mirror-neuron studies to date have been done in nonhuman primates, in the context of actions (8, 10, 26–29). Our study provides insights into how populations of neurons in human PPC mediate shared representations across actual and observed somatosensation, specifically, touch.

We find: 1) that single neurons exhibiting mirror-like properties become increasingly rare as the complexity of the task increases and 2) shared population-level representations can be decomposed into basic building blocks encoded within population-level neural subspaces, which may include classically mirror-like neurons. Combinations of these building blocks through interactions of various subspaces enable human PPC neural populations to represent many forms of touch to different body parts, whether to oneself or applied to another individual. Our findings provide an explanation for how “mirroring” involves heterogenous populations and not exclusively mirror neurons and also connect directly with developing theories of how human-like cognition relies on the nature of mental representations, outlined next.

### Potential Basis for Cognitive Models of the World.

A recent branch of cognitive neuroscience has proposed that human-like learning and thinking are primarily built on the internal models we construct of the world (3, 11). The neural basis for this cognition through internal models framework remains largely unexplored, though neuroimaging points to a role for PPC (11, 32). Our results support this computational architecture while providing granular insight into its neural implementation within human PPC. This framework provides a unifying account of many of our recent results, suggesting that language comprehension, imagination, planning, and perception tap into the same underlying shared internal models (10, 18, 19, 26).

Our results indicate that these internal models are embodied. The tactile responses in the current study have parametrically encoded tactile receptive fields that activate within 60 ms of physical contact (19), consistent with a bottom–up (sensory-driven) role in tactile processing. These responses can be contrasted with the highly selective and long-latency (260 to 400 ms) responses reported for “concept cells” within the medial temporal lobe (33). The fact that tactile imagery (19) and observation engage sensory-like populations suggests that tactile cognition is intricately tied to our somatosensory experiences and argues against purely “symbolist” views of cognition (34). These tactile responses are likely not raw representations of sensory inputs: Neural responses in anterior regions of the PPC are consistent with state estimators that compensate for sensory delays and merge visual and somatosensory inputs (35, 36). Our results suggest that neural populations that help estimate the state of one’s own body may provide inductive biases that constrain and shape our cognitive understanding to be consistent with our own body knowledge.

In prior studies from our lab, we found that the same population of neurons in human PPC also supports different types of actions (attempted, imagined, and executed), by different body parts, as well as spoken and read action verbs. We speculate that a parsimonious and efficient mechanistic possibility is that basic-level information within this population can be recruited in a compositional and generalizable manner to diverse contexts, enabling shared information to support many sensory, cognitive, and motor behaviors. In other words, the basic-level features for touch we identified in this study may belong within shared representational basic-level features that represent an embodied internal model of the world that our brains engage to understand the world around us and guide our behavior.

Within a local neural population, the compositional nature of the population response is structured: Tactile variables related to touch type and body location generalize for any split of the data, consistent with establishing a compositional basis, while the identity of who is being touched does not (Figs. 4, 5). Presumably, models around identity are constructed in varied regions of the temporal cortex, including the medial temporal cortex (37) and the temporal-parietal junction (38). Overall, this pattern of results is consistent with systems-level architectures that construct understanding through the interplay of diverse but interconnected regions (39–44) and suggest that the state of the world is encoded as a distributed population code within and across brain regions. We speculate that if we asked our subject, for instance, to imagine what it would feel like if we pinched her tail (clearly outside her direct experience), we might find that the neural subspace associated with pinches would be activated along with the cortical representation of tails, presumably derived from regions of the temporal cortex.

### Compositionality.

Compositionality captures the basic idea that we construct representations through a combination of more primitive components. For example, a car can be encoded as a combination of wheels, body, engine, seating, steering mechanism, etc. The same elements can be recombined in different ways to form related representations, such as a bus or motorcycle. Two primary approaches have been used to test for compositionality in neural populations: matrix factorization and parts-whole-based approaches. The nature of our stimuli naturally lent itself to the matrix-factorization-based approach (see methods, task description) and may be necessary to ensure adequate behavioral context. For example, the pantomimed gesture of two fingers pressing together may not equate to a “pinch.” To this point, observation of motor movements devoid of goals is insufficient to drive action observation neurons (4). Nonetheless, questions about what constitutes a sufficient stimulus and delving further into the mechanisms of compositionality are exciting directions for future studies.

Compositionality does not imply a specific neural architecture. Concept cells, neurons that respond to a preferred stimulus (e.g. a particular individual) independent of sensory modality or presentation details (e.g., image, written word, sound), can form a compositional basis (45). For example, the concept of “Star Wars” may be formed by an ensemble of cells encoding subconcepts such as “Luke Skywalker”, “Darth Vader”, etc (46). Unlike concept cells, PPC neurons respond to many diverse stimuli in seemingly random ways at an individual cell level (10, 47). Nonetheless, neurons exhibit clear structure at a population level, forming associations between related variables, consistent with an architecture that we have previously defined as partially mixed selectivity. One possibility is that compositionality built on partially mixed representations helps embody or tie our understanding to our lived experiences.

### Relationship to Alternative Accounts of Mirror Neurons.

Alternative explanations for cells that fire to both performed and observed movements have focused on a role for the visual guidance of movement, e.g., by mediating motor imitation, observational learning, or planning in response to the actions of others (48, 49). These are compelling as animals clearly use such observation to guide their motor behavior. In our view, compositional building blocks can provide useful representations that can inform many aspects of behavior. In the motor domain, this may include e.g. action understanding, as well as guiding motor behavior based on the actions of others. In the sensory domain, our findings suggest shared compositional blocks that inform how we experience somatosensations ourselves and understand those that we see around us. A unifying hypothesis is that a mutually shared, universal compositional code underlies both sensory and motor behavior, although this remains to be explored.

### Relevance to BMI.

Numerous clinical trials have shown that individuals with paralysis can use signals from motor regions of the brain to control external devices, such as robotic limbs or computer cursors (18, 50). The underlying brain signals are low-dimensional and roughly encode movement direction smoothly, enabling researchers to collect sufficient data to train a decoding algorithm in a few minutes. Future BMIs could decode high-level concepts, visual imagery, or emotional state. The dimensionality of these signals (e.g., the space of all mental images) is far larger than basic movements. However, if these high-dimensional datasets are encoded using generalizable and low-dimensional basis sets, then the ability to read out these signals may be tractable. To this end, proof-of-concept studies have already demonstrated the ability to decode high-fidelity faces or the semantic content of visual scenes from rich low-dimensional basis sets (17, 51, 52). Our current study provides limited, but consistent, evidence that within the somatosensory domain, basic-level features are combined through population-level subspaces to form higher-level representations. Such findings provide preliminary steps in guiding decoder design for future BMI paradigms.

## Materials and Methods

**Key Resources Table**

**Table:** 

Reagent type or Resource	Source or reference	Identifier
**Software**		
MATLAB	MathWorks, Matlab R2019b	http://www.mathworks.com
Psychophysics toolbox	Psychophysics toolbox PTB3	https://psychtoolbox.org
**Other**		
Neuroport system	Blackrock Microsystems	https://blackrockmicro.com/

## Experimental Model and Study Participant Details

### Subject Details.

All data were recorded from NS, a 62-y-old tetraplegic female participating in a brain-machine interface (BMI) clinical trial. She has a high-cervical spinal cord injury between cervical levels three and four, sustained approximately 10 y prior to the study, and with no preserved sensory or motor function below the shoulder. She was implanted with two 96-channel Neuroport Arrays (Blackrock microsystems model numbers 4382 and 4383) 6 y postinjury, in the left hemisphere. Informed consent was obtained, and she understood the nature, objectives, and potential risks of the surgical procedure and the subsequent clinical studies. All procedures were approved by the Institutional Review Boards (IRBs) at the California Institute of Technology (IRB #18-0401), the University of California, Los Angeles (IRB #13-000576-AM-00027), and Casa Colina Hospital and Centers for Healthcare (IRB #00002372).

### Experimental Setup.

All experiments were conducted at Casa Colina Hospital and Centers for Healthcare. NS was seated in a motorized wheelchair in a well-lit room. A 27-inch LCD monitor was positioned behind NS (visible to the experimenters but not to NS) to cue the experimenters when to deliver tactile stimuli. Cue presentation was controlled by the psychophysics toolbox (Brainard, 1997) for MATLAB (MathWorks).(53)

### Physiological Recordings.

NS was implanted with one Neuroport array at the junction of the intraparietal sulcus and postcentral sulcus, a region we refer to as PC-IP.(19) The other array was implanted in the left superior parietal lobule (SPL). Following surgery, the SPL implant did not function. Only data recorded from PC-IP were used in this study. Both arrays were explanted approximately 2 y after data in this study were collected.

Neural activity recorded from the array was amplified, digitized, and sampled at 30 kHz using a neural signal processor. This system has received Food and Drug Administration (FDA) clearance for <30 d of recordings. We received an investigational device exemption (IDE) from the FDA (IDE #G120096, G120287) to extend the implant duration for the purposes of the BMI clinical study.

Putative neuron action potentials were detected at threshold crossings of −3.5 times the rms of the high-pass filtered (250 Hz full bandwidth signal. Each waveform was made of 48 samples (1.6 ms), with 10 samples prior to triggering and 38 samples after. Single- and multiunit activity was sorted using Gaussian mixture modeling on the first three principal components of the detected waveforms (10). To minimize noise-related effects, we used, as selection criteria, a mean firing rate greater than 0.5 Hz and signal to noise ratio (SNR) >0.5.

## Method Details

### Basic sensory Mirroring Task (BSMT; Relevant for Fig. 1).

This task was performed to establish the shared responsiveness of PPC neurons to actual and observed touch. NS sat facing an experimenter (actor). One experimenter stood behind the actor, and another behind NS. The task involved touch to one of two body parts (cheek, shoulder), to one of two persons (subject, or actor). Touch was provided as rubs performed bilaterally by the experimenter standing behind the person being stimulated, at approximately two rubs per second, for 3 s. Bilateral stimulation was performed (over contralateral stimulation) because in early work from our lab, we noted responses to bilateral touch in the same population of neurons. Bilateral stimulation provided slightly stronger stimulation responses than unilateral stimulation alone. Cheek touches were rubs parallel to the jawline (from cheekbone to chin and back again). Shoulder touches were rubs along the top of the shoulder, from near the neck to the outside of the shoulder and back. The task was performed on six individual recording sessions, with 10 trials per condition. In all, 805 units were recorded, of which 756 met the selection criteria.

### Multidimensional Sensory Mirroring Task (MSMT; Relevant for All Figures Except 1).

This task was performed to understand mechanisms by which neural information is shared across populations of PPC neurons to support actual and observed touch. The basic setup was like the previous task. Here, however, we manipulated three dimensions: two body parts (cheek, shoulder), provided to two persons (NS, actor), in one of four touch-types (pinch, press, rub, tap). As in the BSMT, touch stimuli were provided bilaterally, at approximately two per second, for 3 s. Rubs were as described. Pinches were performed in a nonpainful manner with the thumb, index, and middle fingers. Presses were performed with the index and middle fingers and taps by the tips of the index and middle fingers. Prior to performing the experimental session, we verified that the participant was able to differentiate the different stimuli, whether observed or actual. This task was performed on 8 recording sessions, with 10 trials per condition. In all 806 units were recorded, of which 741 met the selection criteria. A more detailed explanation of this task is provided in *SI Appendix*.

## Quantification and Statistical Analysis

### Linear Analysis (Relevant for Figs. 1*B* and 2*A*).

For each unit, we fit a linear model describing its firing rate as a function of response to each test condition. Response was defined as the mean firing rate between 0.5 after onset of the stimulus phase and ending 0.5 s thereafter. These times were chosen to correspond to the period of time during active tactile stimulation, offset to account for experimenter delays in presenting the stimulus. The baseline was defined as the neural firing rate during the 1 s prior to stimulus presentations. The linear model was computed as

$$FR=\sum_{c}\beta_{c}X_{c}+\beta_{0},$$

where FR is the firing rate, $X_{c}$ is the vector of indicator variables for test condition *c*, $\beta_{c}$ is the estimated scalar weighting coefficient for each condition, and $\beta_{0}$ is a constant offset term. A neuron was considered responsive to a particular condition if the t-statistic for its associated beta coefficient was significant (*P* <0.05, false discovery rate (FDR) corrected for multiple comparisons).

### Discriminability Index (Relevant for Figs. 1*C* and 2*B*).

To quantify how well neural activity can be discriminated from baseline (prestimulus) activity, we used a cross-validated Mahalanobis distance measure. As with the linear analysis described above, the stimulation phase window was defined as 0.5 after onset of the stimulus phase and ending 0.5 s thereafter, and baseline was defined as the 1 s prior to stimulus presentation). The firing rate of all recorded neurons was concatenated into a vector, denoted by A. The firing rate of each neuron during the baseline phase was similarly concatenated to form a vector, denoted by B. Next, a nondimensional distance was computed as

$$DI=\frac{\bar{A}-\bar{B}}{\sqrt{\frac{{\sigma_{A}}^{2}+{\sigma_{B}}^{2}}{2}}},$$

where $\bar{A}$ is the mean of the firing rate vector A, $\bar{B}$ is the mean of the firing rate vector B, $\sigma_{A}$ is the SD of the vector A, and $\sigma_{B}$ is the SD of the vector B.

### Time-Resolved Classification (Relevant for Fig. 2*C*).

Classification was performed using linear discriminant analysis with the following parameter choices: 1) only the mean firing rates differ for unit activity in response to each test condition (covariance of the normal distributions are the same for each condition) and 2) firing rates for each unit are independent (covariance of the normal distribution is diagonal). The classifier took as input a matrix of firing rates for all sorted units. The analysis was not limited to significantly modulated units to avoid “peeking” effects. (54) The analysis was performed independently for each recording session, and results were then averaged across days. In Fig. 2*C*, this analysis was performed in a sliding-time window manner (300 ms each window, stepped at 10 ms intervals), beginning 0.5 s prior to the stimulation onset. Classification performance is reported as the prediction accuracy of a stratified leave-one-out cross-validation analysis.

### Correlation (Relevant for Fig. 1*F*).

We performed cross-validated correlation to compare the neural representations of various test conditions (stimulus presentations) against each other in a pairwise manner. We quantified the neural representations as a vector of firing rates, one vector for each condition with each vector element summarizing the response of an individual unit. Neural activity was summarized as the mean firing rate during the stimulation phase window, defined as before (0.5 s after onset of the stimulus phase to 0.5 s after it ended). Firing rate vectors were constructed by averaging the responses across 50 to 50 splits of trial repetitions. The mean responses across different splits were correlated within and across conditions, then the splits were regenerated, and the correlation computed 250 times. The within-condition correlations assist in our interpretation of the across-sensory field correlations by allowing us to quantify the theoretical maxima of the similarity measure (e.g., if the within-condition correlation is measured at 0.6, then an across condition of 0.6 suggests the maximal level of similarity as allowed by the trial-to-trial variability of the signal).

### Event-Related Averages (Relevant for Figs. 1*E* and 2 *D*–*F* and *SI Appendix*, Fig. S2).

For each unit, neural activity was averaged within 750 ms intervals starting from 0.5 s prior stimulation onset, stepping to 2.5 s after, in 100 ms step intervals. Responses were grouped by condition, and a mean and SEM were computed for each time window and for each condition.

### Modeling the Single-Neuron Response Properties to Various Test Conditions (BSMT) (Relevant for Fig. 1*D*).

This analysis was performed to understand how individual neurons responded to four formats: actual cheek touch (Ac), actual shoulder touch (As), observed cheek touch (Oc), and observed shoulder touch (Os). Various possibilities exist. For example, the neuron might respond to actual touch to both body parts but not to any observed touch. Alternatively, it could respond to both actual and observed touch to the one body part but not to the other. We can model the firing rate for a given unit as

$$fr=\alpha ∙A_{c}+\beta ∙A_{s}+\gamma ∙O_{c}+\delta ∙O_{s},$$

where fr is the firing rate for the unit, $A_{c}$, $A_{s}$, $O_{c}$, $O_{s}$ are the four formats, and α, β, γ, and δ are the weighting coefficients for each sensory field, respectively. If the unit does not respond to a sensory field, then the dot product of the unit’s weighting coefficient and the sensory field collapses to a scalar value. If a unit responds to two formats in a congruent manner, then the weighting coefficient for these two formats will be identical. For the analysis, we allowed a weighting coefficient to be either 0 or 1, such that across 4 formats, there are a total of 16 possible models for each neuron. We fit the parameters of each of the 16 models using standard linear regression techniques (see above), and the results were compared. As selection criteria to evaluate the “best” model from all candidate models, we used the BIC and cross-validated coefficient of determination (cvR^2^). The models were grouped according to four categories: invariant (in which the weighting coefficient was identical across all formats), body part specific (in which the weighting coefficient was invariant for matched body parts, but not for mismatched body parts), person specific (in which the weighting coefficient was invariant for touch to the same person), or idiosyncratic (all other combinations).

### Modeling the Single-Neuron Response Properties to Various Test Conditions (MSMT) (Relevant for Fig. 3 and *SI Appendix*, Fig. S3 and S4).

This analysis is like the earlier modeling analysis for the BSMT, except it has been expanded to accommodate for more test conditions. To understand the breakdown of individual units that create the population response, we first defined four formats: actual cheek touch (Ac), actual shoulder touch (As), observed cheek touch (Oc), and observed shoulder touch (Os). An individual neuron could respond to one or more formats. If it responds to more than one sensory field, it could respond with a congruent selectivity pattern (SP; the precise pattern of responses) to each of the four touch types (pinch, press, rub, tap) within the sensory field, or with an incongruent SP. Across the four formats, the firing rate for a given unit can be described mathematically as

$$fr=\alpha ∙A_{c}+\beta ∙A_{s}+\gamma ∙O_{c}+\delta ∙O_{s},$$

where fr is the firing rate for the unit, $A_{c}$, $A_{s}$, $O_{c}$, $O_{s}$ are the four formats, and α, β, γ, and δ are the weighting coefficients for each sensory field, respectively. If the unit does not respond to a sensory field, then the dot product of the unit’s weighting coefficient and the sensory field collapses to a scalar value. Within this type of a linear model, if a unit responds to formats with an identical SP, then the weighting coefficient for all those formats will have an identical weighting coefficient. In all, there are 51 unique models for all the ways in which SPs can be expressed across formats.

To determine how SPs compared across formats, we fit the parameters of each of the 51 models using standard linear regression techniques (see above), and the results were compared. As selection criteria to evaluate the best model from all candidate models, we used the BIC and cross-validated coefficient of determination (cvR^2^). Results are summarized as the number of units that are best described by a particular model.

### Demixed Principal Components Analysis (Relevant for Fig. 4), and Generalizability Analysis (Relevant for Fig. 5).

Descriptions of both these analyses, specific to the two figures mentioned, are described in detail in *SI Appendix*.

## Supplementary Material

Appendix 01 (PDF)

Movie S1. **Example of a neuron demonstrating a mirror-like response**. This example neuron activated when NS felt a touch to her outer shoulder but did not activate when she felt a touch to her inner shoulder. This represents an example of the specificity of neural response. In addition, the neuron activated when NS visually observed a touch to the experimenter’s outer shoulder but did not activate when observed a touch to the experimenter’s inner shoulder. The fact that response properties of experienced and observed tactile sensations were similar demonstrates the congruency of the neural response. The combined properties of specificity and congruency are the hallmark of a mirror-like response.

Movie S2. **Second example of a neuron demonstrating a mirror-like response**. This example neuron activated when NS felt a touch on her cheek and when she observed a touch on another person's cheek, but not when she observed a touch on the cheek of a Styrofoam head. The combined properties of congruency of response for felt and observed touch and specificity to touch to a human subject are consistent with a mirror-like response.

## Data Availability

Data and code used for analysis are available on Zenodo (55) and GitHub (56). For any questions relating to code or data, the lead author can be contacted with reasonable request.
