# Supplementary material for: Population encoding of observed and actual somatosensations in the human posterior parietal cortex
Source: Proc Natl Acad Sci U S A. 2024 Dec 30;122(1):e2316012121. doi: 10.1073/pnas.2316012121 (PMC11725854; doi:10.1073/pnas.2316012121)
Supplement: Supplementary file 1 — Appendix 01 (PDF) [file pnas.2316012121.sapp.pdf]

PNAS

# Supporting Information for

## Population encoding of observed and actual somatosensations in human posterior parietal cortex

Srinivas Chivukula, Tyson Aflalo, Carey Zhang, Emily R. Rosario, Ausaf Bari, Nader Pouratian, Richard A. Andersen

Corresponding authors: Richard Andersen
Richard.andersen@vis.caltech.edu

**This PDF file includes:**

Figures S1 to S5

**Other supporting materials for this manuscript include the following:**

Movies S1 to S2
Statistical Analysis for Figures 4 and 5 in Main Manuscript File

**Supplementary Figures**

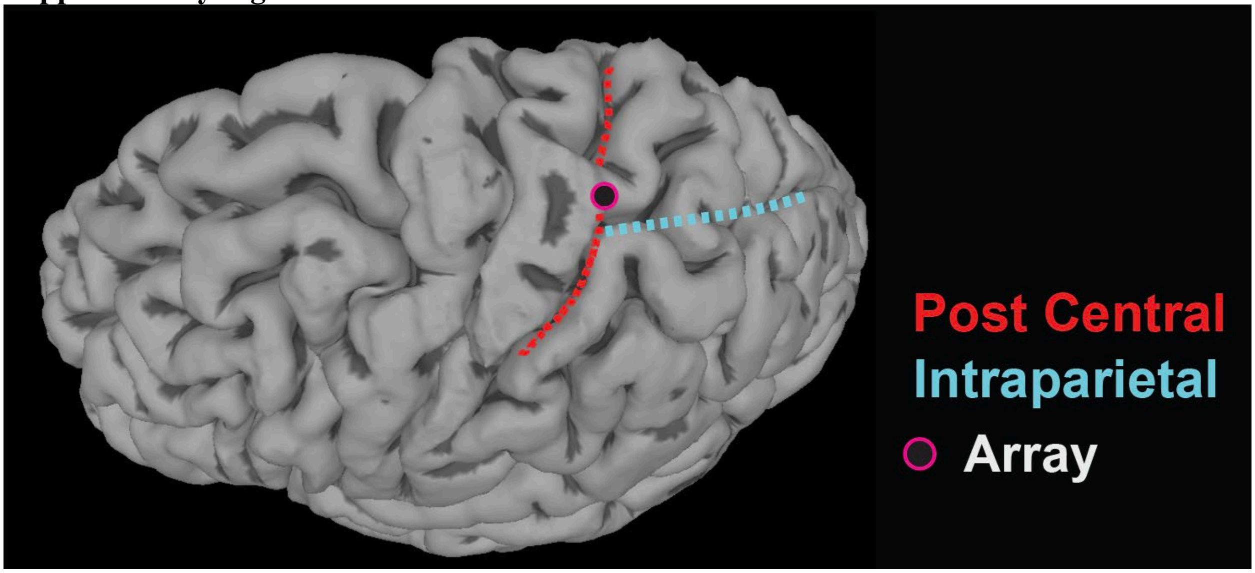


**Figure S1: Microelectrode array implantation location. Related to Figures 1&2.** Individual participant anatomy with the location of the microelectrode array implant.

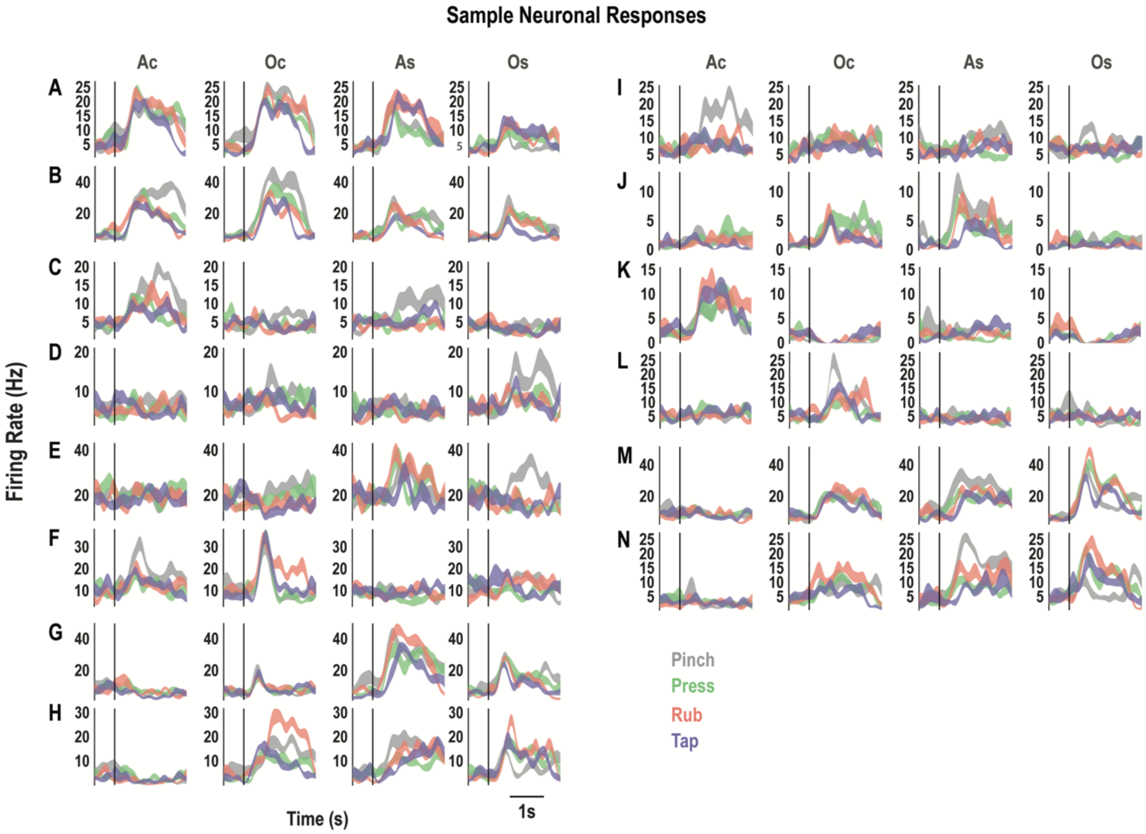


**Figure S2. Individual neurons exhibit complex and variable response patterns. Related to figure 2.**

**A-N**, Firing rate as a function of time for example neurons illustrating diverse responses to tactile stimuli across the different sensory fields (columns; Ac, actual cheek touch; Oc, observed cheek touch; As, actual shoulder touch; Os, observed shoulder touch). Within each panel, the neural response to each of the four touch types is shown (colors as in legend), as the mean firing rate (y-axis) ± SEM, n=10 trials, as a function of time (x-axis). Ac, actual cheek; Oc, observed cheek; As, actual shoulder; Os, observed shoulder; Hz, hertz; s, seconds.

**A**

| Body-Part mirror | Invariant | Person | Body-Part other | Three field congruent | Two field congruent | One field | Idiosyncratic |
|---|---|---|---|---|---|---|---|
| Ac=Oc | Ac=As=Oc=Os | Ac=As | Ac=Oc&As | Ac=As=Oc | As=Oc | Ac | As&Os |
| As=Os | | Oc=Os | Ac=Oc&Os | Ac=Oc=Os | Ac=Os | As | Oc&Os |
| | | Ac=As&Oc | As=Os&Ac | Ac=As=Os | As=Oc&Os | Oc | Ac&As |
| | | Ac=As&Os | As=Os&Oc | As=Oc=Os | As=Oc&Ac | Os | Ac&Oc |
| | | Oc=Os&Ac | As=Oc&Ac&Oc | Ac=As=Oc&Os | Ac=Os&As | | As&Oc |
| | | Oc=Os&Ac&As | Ac=Oc&As&Os | Ac=Oc=Os&As | Ac=Os&Oc | | Ac&Os |
| | | Ac=As&Oc&Os | Ac=Oc&As=Os | Ac=As=Os&Oc | Oc=Os&As | | As&Oc&Os |
| | | As=As&Oc=Os | | As=Oc=Os&Ac | Ac=Os&As&Oc | | Ac&Oc&Os |
| | | | | | As=Oc&Ac&Os | | Ac&As&Os |
| | | | | | Ac=Os&As=Oc | | Ac&As&Oc |
| | | | | | | | Ac&As&Oc&Os |

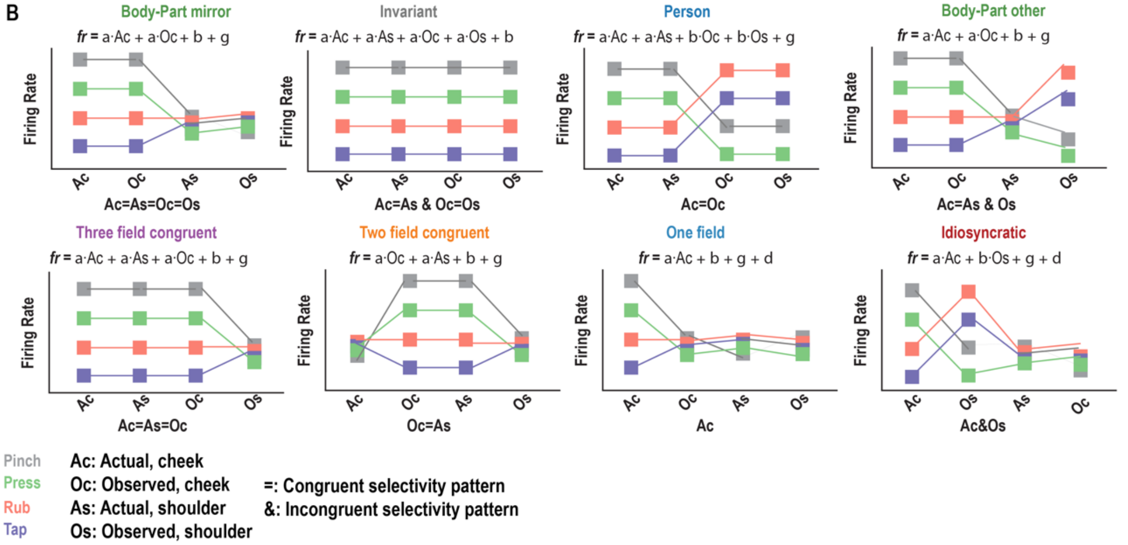


**Figure S3. Schematic illustration describing congruency in selectivity patterns across sensory fields. Related to figure 3. A**, All 51 possible linear models that could describe an individual neuron's response (selectivity pattern, SP) to the four different touch types. The '=' symbol denotes congruency in SP between the sensory field listed before and after the symbol, and the '&' symbol denotes incongruent SPs. The 51 possible models are grouped into 8 categories for interpretative purposes, labeled accordingly. Ac, actual cheek touch; As, actual shoulder touch; Oc, observed cheek touch; Os, observed shoulder touch. **B**, Schematic illustrations of each category of the 51 possible linear models. Each panel shows one example case for the columns shown in panel A, as labeled underneath each panel. The examples illustrate cases in which the SP is congruent between actual and observed touch to the cheek but unresponsive to shoulder touch (body-part mirroring), the SP is congruent across all sensory fields (invariant), and congruent within-person (i.e., for actual cheek and actual shoulder touch) but incongruent from actual to observed touch (person). The mathematical description of each model is shown above each illustration. In the analysis, congruency is operationalized by using the same linear-model coefficients to describe responses to multiple sensory fields. Incongruency uses distinct linear model coefficients. An abbreviated name for the model is shown below each panel. The '=' symbol denotes congruency in SP between the sensory field listed before and after the symbol, and the '&' symbol denotes incongruent SPs across sensory fields. See figure S3 for a full list of models and examples for each category of behavior. Ac, actual cheek touch; As, actual

shoulder touch; Oc, observed cheek touch, Os, observed shoulder touch; fr, firing rate.

**Description of the eight summary models:** The eight models can be understood as follows: Body Part Mirror describes models in which there is unambiguous congruency between actual and observed touch, specific to a body part. Invariant describes the case in which neural responses are congruent across all sensory fields. Person describes neurons where there is a congruent response to the different body parts within an individual, but incongruency between actual and observed touch. Body part other describes neurons with congruency between actual and observed touch to one body part, but some form of incongruency between body parts thus creating ambiguity about the stimulus at the single neuron level. Three and two field congruent describes neurons showing congruency across 3 or 2 of the 4 sensory fields, but are not well-described by the other models (e.g., an example would be congruency between actual shoulder and observed cheek responses.) One field describes neurons that show discriminable responses between touch types for only one field. Idiosyncratic describes neurons that show incongruent responses to two or more sensory fields.

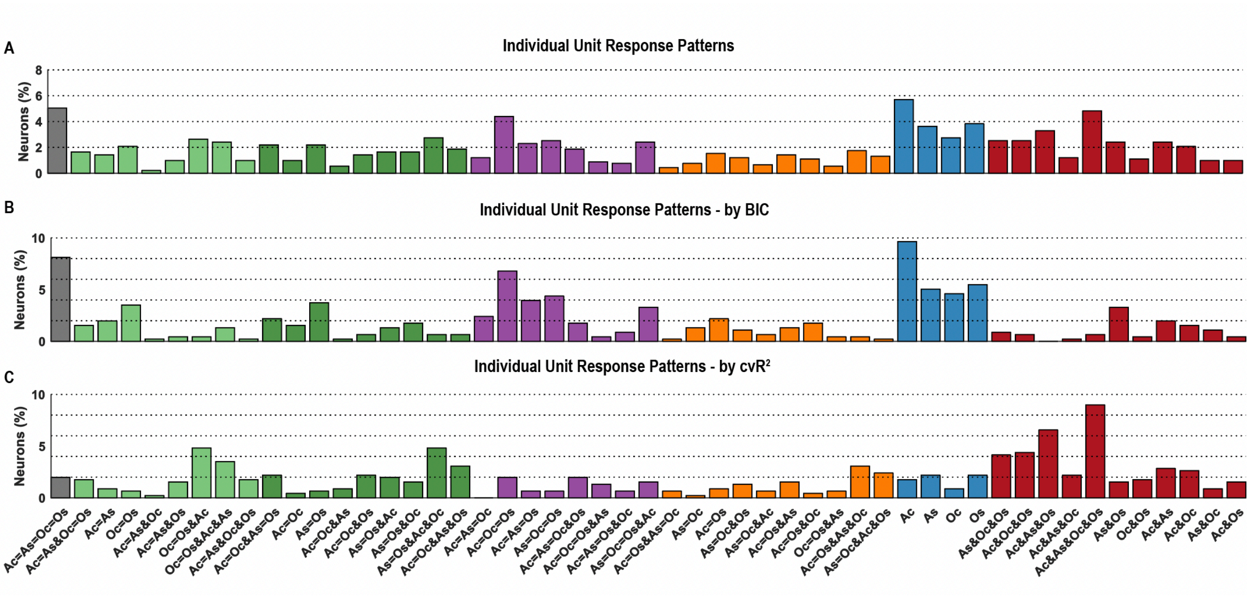


**Figure S4. Categorization of all single-units into 51 possible patterns of correspondence across sensory fields. Related to figure 3.**

**A,** Histogram showing the percentage of PPC neurons that behaved according to each of the 51 possible linear models. The abbreviated form of the model is listed below each bar. As in Figure S3, the '=' symbol denotes congruency in SP between the sensory fields listed before and after the symbol, and the '&' symbol denotes incongruency. Bars are color-coded by category (see figure 3). Ac, actual cheek touch; As, actual shoulder touch; Oc, observed cheek touch, Os, observed shoulder touch. **B-C,** Similar to A except categorizations are shown for Bayesian information criteria (BIC) and the cross-validated coefficient of determination ($cvR^2$) separately.

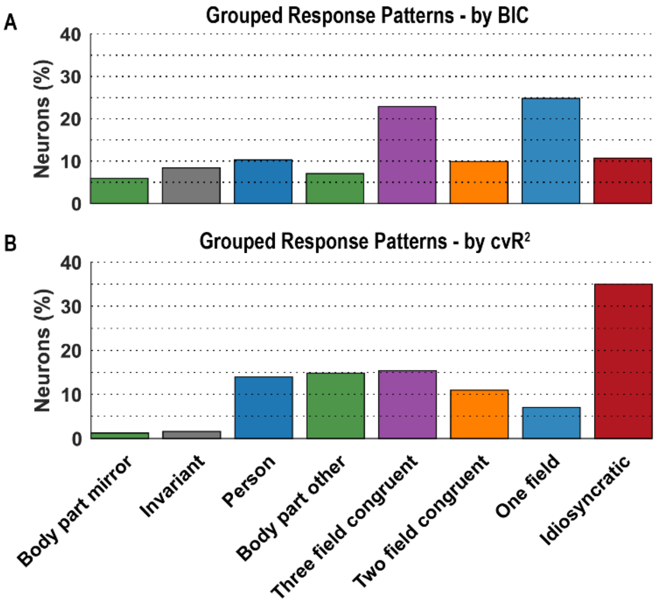


**Figure S5. Summary of categorizing single-units into 51 possible patterns of correspondence across sensory fields based on BIC and $cvR^2$. Related to figure 3.**

**A,** Histogram showing the percentage of PPC neurons that behaved according to each of the 8 categories of linear models (see Fig S3). Here the breakdown is shown based on using the Bayesian Information Criterion (BIC) as the metric for evaluating which of the 51 models best matched each neuron's behavior. The category name is listed below each bar. **B**, Similar to A, except here the breakdown is based on the cross-validated coefficient of determination $(cvR^2)$.

**Movie S1: Example of a neuron demonstrating a mirror-like response.** This example neuron activated when NS felt a touch to her outer shoulder but did not activate when she felt a touch to her inner shoulder. This represents an example of the specificity of neural response. In addition, the neuron activated when NS visually observed a touch to the experimenter's outer shoulder but did not activate when observed a touch to the experimenter's inner shoulder. The fact that response properties of experienced and observed tactile sensations were similar demonstrates the congruency of the neural response. The combined properties of specificity and congruency are the hallmark of a mirror-like response.

**Movie S2: Second example of a neuron demonstrating a mirror-like response.** This example neuron activated when NS felt a touch on her cheek and when she observed a touch on another person's cheek, but not when she observed a touch on the cheek of a Styrofoam head. The combined properties of congruency of response for felt and observed touch and specificity to touch to a human subject are consistent with a mirror-like response.

## QUANTIFICATION AND STATISTICAL ANALYSIS

*Multidimensional sensory mirroring task.* The use of three factors was essential to enable testing for compositionality in the neural code. There are two primary approaches that have been used to test for compositionality in neural populations: matrix factorization and parts-whole based approaches. In the matrix factorization approach, population activity is measured while the participant views complex stimuli composed of varied combinations of different constituent elements (1-4). The resulting activity patterns are then subjected to some form of matrix decomposition to test whether the stimuli are explainable as combinations of the constituent elements. For example, a stimulus set may consist of images of young men and women and old men and women. The resulting brain activity would then be decomposed to test whether neural responses can be represented as combinations of age and gender dimensions. In the parts-whole approach, population activity is measured while the participant views stimuli of the parts and the whole separately (5). Brain responses evoked by the parts are then combined to see if they predict the brain response to the whole. For example, a stimulus set may consist of the words "old," "man," and "grandpa." Response to "old" and "man" are then summed to see if they predict "grandpa." This latter approach has intuitive appeal as it has a direct relationship to the underlying theory – the whole is the sum of its parts. However, representing the parts is not trivial for certain classes of stimuli. One cannot simply show an image of "old"; there must be an embodiment – an image of something old. Additionally, it is unclear whether parts, devoid of their surrounding context, would or should be processed as such. For example, the pantomimed gesture of two fingers pressing together may not equate to a "pinch." To this point, observation of motor movements devoid of goals are insufficient to drive action observation neurons (6). These latter cases naturally lend themselves to the matrix factorization approach – e.g. to show stimuli of the "old something" and use an appropriate analysis technique to separate the neural signature of "old" from the neural signature of the "something." This approach carries the possibility of overfitting the data, finding a separation that is not a true generalizable feature of the data. To mitigate against this possibility, it is essential to record a dataset that allows any identified structure to be validated against data that was not used to train the algorithm. Such an approach requires a sufficient number of factors, at minimum three, so that the matrix decomposition can across at minimum two factors and tested across varied levels of the third factor.

*Demixed principal components analysis (relevant for Figure 4)* To test for generalization and compositionality, we wished to 1) perform a supervised dimensionality reduction analysis that used a portion of the data to find a matrix projection from the high-dimensional neural population data into a low-dimensional compositional latent space and 2) test whether the discovered matrix recovered the same latent dimensions when applied to test data (see figure 4a). We used demixed-principal component analyses (dPCA) as our supervised-dimensionality reduction algoriothm. dPCA is an analysis technique that decomposes population neural data along user-defined neural dimensions (marginalizations) that capture variance related to task variables (7). This decomposition provides insight into the structure in neural data as it relates to the experimentally

manipulated variables. We used all sorted units in this analysis. dPCA takes as input a matrix that describes firing rates to each of the test conditions for each trial as a function of time. Neural activity was averaged within 750 ms intervals starting from 0.5 s prior to the onset of the stimulation phase, stepping to 0.25 s after the stimulus offset, in 50 ms step intervals, to the time of stimulus offset. In our current study, we were interested in understanding how much of the population variance was explained by independent dimensions (i.e., body-part, touch type, and person being touched) as well as by interaction terms (touch type x body-part, body-part x person, touch type x person).

Our interpretation of the dPCA analysis assumes that neural subspaces associated with task variables are generalizable, capturing aspects of the data that would apply to different contexts and stimuli. For example, the neural subspace associated with the main effect of body location should code for that body location whether touch is actual or observed, and for all forms of touch. In order to validate whether the discovered subspaces are indeed generalizable, perform the dPCA analysis on a subset of the data and test on the held-out data. To this end, we trained a two-factor dPCA on multiple levels of two dimensions of the data for a single fixed value of the third dimension. We then test the subspaces using entirely held-out data, from the alternate value of the third dimension. The train and test splits are shown in figure 4. If the dPCA accurately accounts for the neural variability in the held-out data, then we can interpret the subspaces as being a generalizable feature of the neural population.

*Generalizability analysis (relevant for Figure 5).* The mirror mechanism is proposed to link what we see with what we intend or feel. Based on our previous data within this PPC substrate, we hypothesized that the mirror mechanism is one manifestation of a broader computational strategy by which PPC neurons generalize across task dimensions (behavioral contexts). To test this hypothesis, we train a linear model to identify a neural subspace that discriminates values along one dimension for fixed values of the other two dimensions. Then, we test whether this subspace allows similar discrimination for alternate values of the fixed dimensions. For example, we train the model to discriminate touch-types (dimension 1, pinch versus press), for fixed body part (dimension 2, e.g., cheek) and person (dimension 3, e.g., NS) and test the ability to discriminate touch-type when switching body part, person, or both body part and person. This analysis provides a population-level generalization of the basic mirror neuron test, testing for both specificity (operationalized by finding the population response that discriminates between two conditions) and congruency (testing whether this population response is congruent between (generalizes across) self and other, or potentially other task dimensions.) If the mirror-mechanism is the dominant motif that determines population-level encoding, then we would predict preferential congruency/generalization when matching body locations and touch-types between self and other. Otherwise, under a more general mechanism for shared encoding, we would expect generalizable information to be a ubiquitous phenomenon.

We quantified the neural response to each condition as a vector of firing rates, one vector for each condition, with each element in each vector summarizing the response of an individual unit. All sorted units were used in this analysis. As in other analyses, neural

activity was summarized as the mean firing rate beginning 0.5 s after onset of stimulation phase and ending 0.5 s after its conclusion. Next, we identified population-level neural subspaces that optimally differentiate between pairs of conditions (training conditions) and tested how well these subspaces differentiate between pairs of test conditions. For example, in Figure 5A, we identified a subspace that optimally differentiates between the two touch types, pinch and press, during actual touch the participant's cheek and asked: how well does this subspace also differentiate between touch-types when observing them applied to the cheek? To create the subspaces, we linearly regressed (using partial least squares regression) the vectors for the pair of training conditions, such that the cross-validated Mahalonbis distance between the two conditions was maximized. We then used this model to project the held-out test data into the same subspace and computed the Mahalanobis distance between conditions. This computed distance was normalized to the cross-validated distance of the training data and thus the resulting metric expresses how well the test conditions are separable relative to the training conditions. In this way, the analysis is able to tell us how well a neural subspace that optimally distinguishes the training conditions is able to apply o the test conditions. A value of 1 indicates that the neural subspace that maximally separates the training data (e.g., measured during actual touch) perfectly generalizes to the held-out test data (e.g. measured during observation). This basic computation was performed in reverse as well, such that in this example, a subspace was created that optimally differentiated between ***observed*** cheek pinch and presses, and tested to identify separability between ***actual*** cheek pinches and presses. The results in both directions were averaged and recorded as the normalized generalizability of information for presentation purposes (in this example case, the generalizability of information separating touch-types across ***actual*** and ***observed*** touch). The generalizability was computed for each day independently and averaged across recording sessions. Confidence intervals were estimated using a bootstrap procedure.

**References**


1. C. K. Machens, R. Romo, C. D. Brody, Functional, But Not Anatomical, Separation of "What" and "When" in Prefrontal Cortex. *Journal of Neuroscience* **30**, 350-360 (2010).
2. L. Chang, D. Y. Tsao, The Code for Facial Identity in the Primate Brain. *Cell* **169**, 1013-1028 e1014 (2017).
3. F. R. Willett *et al.*, Hand Knob Area of Premotor Cortex Represents the Whole Body in a Compositional Way. *Cell* **181**, 396-409 e326 (2020).
4. E. Bizzi, V. C. Cheung, A. d'Avella, P. Saltiel, M. Tresch, Combining modules for movement. *Brain Res Rev* **57**, 125-133 (2008).
5. S. G. Baron, D. Osherson, Evidence for conceptual combination in the left anterior temporal lobe. *Neuroimage* **55**, 1847-1852 (2011).
6. G. Rizzolatti, C. Sinigaglia, The mirror mechanism: a basic principle of brain function. *Nat Rev Neurosci* **17**, 757-765 (2016).
7. D. Kobak *et al.*, Demixed principal component analysis of neural population data. *Elife* **5** (2016).
